# One-Year Clinical Outcomes of Minimal-Invasive Dorsal Percutaneous Fixation of Thoracolumbar Spine Fractures

**DOI:** 10.3390/medicina58050606

**Published:** 2022-04-27

**Authors:** Babak Saravi, Sara Ülkümen, Sebastien Couillard-Despres, Gernot Lang, Frank Hassel

**Affiliations:** 1 Department of Orthopedics and Trauma Surgery, Medical Centre—Albert-Ludwigs-University of Freiburg, Faculty of Medicine, Albert-Ludwigs-University of Freiburg, Hugstetterstrasse 55, 79106 Freiburg, Germany; sara.uelkuemen@hotmail.de (S.Ü.); gernotmichaellang@gmail.com (G.L.); 2 Department of Spine Surgery, Loretto Hospital, 79100 Freiburg, Germany; frank.hassel@rkk-klinikum.de; 3 Institute of Experimental Neuroregeneration, Spinal Cord Injury and Tissue Regeneration Center Salzburg (SCI-TReCS), Paracelsus Medical University, 5020 Salzburg, Austria; s.couillard-despres@pmu.ac.at; 4 Austrian Cluster for Tissue Regeneration, 1200 Vienna, Austria

**Keywords:** percutaneous fixation, minimal-invasive, thoracolumbar fractures, spine, retrospective cohort study, prediction, PROMs

## Abstract

Introduction: Minimal-invasive instrumentation techniques have become a workhorse in spine surgery and require constant clinical evaluations. We sought to analyze patient-reported outcome measures (PROMs) and clinicopathological characteristics of thoracolumbar fracture stabilizations utilizing a minimal-invasive percutaneous dorsal screw-rod system. Methods: We included all patients with thoracolumbar spine fractures who underwent minimal-invasive percutaneous spine stabilization in our clinics since inception and who have at least 1 year of follow-up data. Clinical characteristics (length of hospital stay (LOS), operation time (OT), and complications), PROMs (preoperative (pre-op), 3-weeks postoperative (post-op), 1-year postoperative: eq5D, COMI, ODI, NRS back pain), and laboratory markers (leucocytes, c-reactive protein (CRP)) were analyzed, finding significant associations between these study variables and PROMs. Results: A total of 68 patients (m: 45.6%; f: 54.4%; mean age: 76.9 ± 13.9) were included. The most common fracture types according to the AO classification were A3 (40.3%) and A4 (40.3%), followed by B2 (7.46%) and B1 (5.97%). The Median American Society of Anesthesiologists (ASA) score was 3 (range: 1–4). Stabilized levels ranged from TH4 to L5 (mean number of targeted levels: 4.25 ± 1.4), with TH10-L2 (12/68) and TH11-L3 (11/68) being the most frequent site of surgery. Mean OT and LOS were 92.2 ± 28.2 min and 14.3 ± 6.9 days, respectively. We observed 9/68 complications (13.2%), mostly involving screw misalignments and loosening. CRP increased from 24.9 ± 33.3 pre-op to 34.8 ± 29.9 post-op (*p* < 0.001), whereas leucocyte counts remained stable. All PROMs showed a marked significant improvement for both 3-week and 1-year evaluations compared to the preoperative situation. Interestingly, we did not find an impact of OT, LOS, lab markers, complications, and other clinical characteristics on PROMs. Notably, a higher number of stabilized levels did not affect PROMs. Conclusions: Minimal-invasive stabilization of thoracolumbar fractures utilizing a dorsal percutaneous approach resulted in significant PROM outcome improvements, although we observed a complication rate of 13.2% for up to 1 year of follow-up. PROMs were not significantly associated with clinicopathological characteristics, technique-related variables, or the number of targeted levels.

## 1. Introduction

The thoracolumbar spine is the most common region affected by traumatic spinal column fractures [1]. This area has an essential role in spine biomechanics as it represents the transition zone from the rigid thoracic spine, which is less mobile due to its connection to the ribs and sternum, to the more flexible lumbar spine [2]. The global annual incidence of thoracolumbar spine fractures is about 30/100,000 people when osteoporotic fractures are included [3]. In the USA, 17,000 new spinal cord injury cases occur each year, and more than 250,000 patients are currently living with permanent deficits [4]. There is a trend of increased fracture rates in the elderly population, especially in developed countries, while an increase in motor vehicle accidents as a significant etiological factor can be found in developing countries [3]. Although thoracolumbar fractures were mainly found in younger ages (15–29 years old) in the last century, the median age is currently 35 [5].

Fractures of the thoracolumbar region can be very variable and involve simple non-displaced fractures to more complex fracture–dislocation. Up to 75% of spine fractures occur in the T10-L2 area [6]. In 40% to 80% of the cases, disruptions of the thoracolumbar spine are often a result of high-energy injuries by an axial load, with or without flexion force, affecting the anterior and middle columns of the vertebral body [1]. The majority are the result of motor vehicle accidents and falls [3]. Blunt trauma is associated with an incidence of 1.9% thoracic fractures [4]. Notably, up to 27% of thoracolumbar junction injury cases develop neurological deficits, resulting in a substantial socioeconomic burden [7]. Numerous spine injury classification systems have been introduced to date, while for the thoracolumbar spine, fracture classification is frequently done utilizing the AOSpine Trauma Classification system for spinal trauma [8].

All treatment approaches in affected patients aim to protect or restore neurologic function and stabilize the column to prevent further instability, potentially resulting in pathological collapse or deformity of the spinal column. Validated measures in spinal care are necessary for clinicians to evaluate the effectiveness of therapies. Such measures are also critical for researchers to quantify patients’ health-related quality of life for scientific investigations. Improving patient-related outcome measures (PROMs) is considered a major objective in spinal surgery [9]. In particular, function loss and pain can impair life quality and significantly affect PROMs. While treating fractures without neurological deficits is still controversial, vertebral fractures associated with neurologic deficits should undergo surgical decompression and stabilization [10,11]. Classically, surgical treatment consisted of internal fixation using open approaches [11]. Assaker et al. (2004) were the first to report on the management of vertebral fractures with percutaneous fixation [12]; this was followed by numerous studies demonstrating good radiologic and clinical outcomes with posterior fracture reduction and instrumentation [11]. 

Although several studies have investigated the clinical application of percutaneous fixation in the treatment of thoracolumbar fractures, there is still little evidence from studies investigating the predictive factors influencing PROMs in affected patients. Evaluation of relevant associated factors can help to build predictive models, modify treatment guidelines, and improve PROMs, following the fundamentals of precision medicine. Hence, we reviewed all cases in our institution treated with a minimal-invasive dorsal percutaneous fixation of thoracolumbar spine fractures to shed light on this topic.

## 2. Materials and Methods

### 2.1. Study Design

We performed a retrospective cohort study including consecutive patients treated with minimal-invasive dorsal percutaneous fixation of thoracolumbar spine fractures since 2014 at the Department of Spine Surgery, Loretto-Hospital Freiburg in Germany. This study defined thoracolumbar spine fractures as index fractures found between segment level Thoracic(Th)7-Lumbar(L)4. The rationale for performing the percutaneous fixation in patients with non-displaced stable fractures was that patients who started conservative treatment with a brace then showed a loss of height of the fracture and/or couldn’t cope with the pain. This retrospective observational study was approved by the local Ethics Committee Freiburg, Germany [Number: 116/200]. Written informed consent to participate in observational studies was obtained from each patient.

Patients were stabilized using either the Percusys^®^ screw-rod system (joimax^®^ GmbH, Karlsruhe, Germany) or the MIS Z-Pedicle Screw System (Z-Medical, Tuttlingen, Germany). Both systems are technically identical; thus, the term screw-rod system (SRS) is used to describe the intervention. Polyaxial or quattroaxial screws were utilized for the Z-Medical system in our institution, whereas the Percusys system consists of solely polyaxial screws. The preoperative situation of the SRS group was the baseline control for all outcome measures, as we did not apply a case-control design.

### 2.2. Data Handling

Patients were collected from the in-house patient information system, utilizing the following inclusion criteria to extract the data: (1) thoracolumbar spine fracture and (2) treatment with one of the aforementioned pedicle-screw systems from January 2014 to Janurary 2021. Hereafter the data were extracted into a predefined datasheet. Data were pseudonymized utilizing a code generated with the “encode” command in Stata Statistical Software Release 15 (StataCorp. 2011, College Station, TX, USA).

Three main groups of variables were included in the data extraction form. The surgery-related and clinical factor variable group included operation time (OT), length of hospital stay (LOS), number of stabilized levels (NSL), the American Society of Anesthesiologists (ASA) physical status classification, the Arbeitsgemeinschaft für Osteosynthesefragen (AO) classification, and complication rates. This group also contained demographic data for descriptive statistics (e.g., sex, age, etc.). The AO classification divides fractures into three categories: compression (group A), distraction (group B), and translation or rotation (group C) [13]. Groups A through C represent progressively increasing injury severity and instability. The laboratory variable group included C-reactive protein (CRP) and white blood cell count (WBS). Patient-related outcome measures (PROMs) were targeted as the dependent variables for outcome evaluations and included the German version of the Oswestry Disability Index (ODI) [14], Core Outcome Measures Index (COMI) [15], the Numeric Rating Scale (NRS) of leg and back pain [16], and the eQ-5D health questionnaire [17]. Patients had follow-ups at regular time points to evaluate treatment success, and PROMs were assessed preoperatively and 3 weeks and 1 year postoperatively. Patient data were screened for the following complications: new-onset sensorimotor deficits, hematomas, postoperative instability and fracture, screw loosening, and misalignment.

### 2.3. Statistical Analysis

Data were first evaluated with descriptive statistics. Means are shown with their standard deviation, whereas medians are reported with the interquartile range. Cohort characteristics and PROMs for the three time points (preoperatively and 3 weeks and 1 year postoperatively) were then compared utilizing the Friedman test and Wilcoxon signed-rank test to account for the repeated measures design, and retrieved *p*-values were adjusted by the Bonferroni correction. Bar charts with mean ± standard deviation were created in GraphPad Prism Software version 8.2.1 (GraphPad Software, Inc., San Diego, CA, USA). Thereafter, we checked for variables showing an association with the PROMs utilizing a multivariate linear mixed effect model that includes both fixed and random effects to account for the within-participant repeated measures outcome evaluation. In addition, PROMs were dichotomized (“clinically significant improvement” versus “no clinically significant improvement”), using at least 25% improvement of the postoperative situation as the cut-off value for a minimally important clinical difference to explore significant relationships of study variables [18]. For the eq5D and NRS, cut-off values of 0.040 [19] and 2.0 [20] were assumed to correspond to a minimally important clinical difference. Thereafter, Cramer’s V (larger than 2 × 2 contingency table), phi coefficient (2 × 2 contingency table), and Pearson’s chi-squared were applied to investigate the possible relationship between the PROM improvement status and study variables. Interpretation of Cramer’s V and phi coefficient was made as follows: very strong correlation (>0.25), strong correlation (>0.15), moderate correlation (0.10), weak correlation (>0.05), no or very weak correlation (>0) [21]. Further, logistic regression analyses were performed to evaluate predictors for the dichotomized PROMs as dependent variables, and the Odds Ratio (OR) and the 95% confidence interval (95% CI) were retrieved from the regression models. A *p*-value <0.05 was considered significant. Statistical analyses were conducted in Stata Statistical Software Release 15 (StataCorp. 2011, College Station, TX, USA) and SPSS v27 (IBM, Armonk, NY, USA).

## 3. Results

### 3.1. Cohort Characteristics

**Figure 1:**
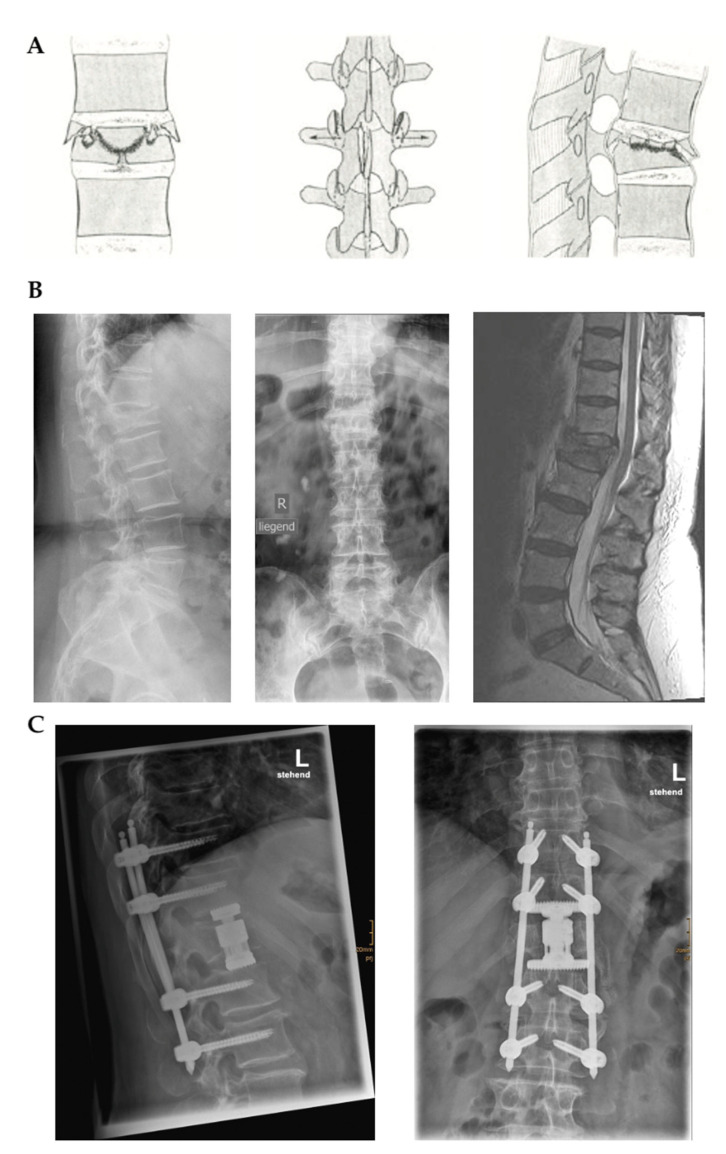
Illustration of one case of the cohort with an A3 cranial burst split fracture (**A**,**B**). Percutaneous stabilization at level Th11-L3, nucleotomy at Th12/L1 and L1/L2, corporectomy, and vertebral body replacement at L1 (**C**) were used to manage the fracture.

The cohort consisted of n = 68 patients (male: 31, female: 37). The youngest patient in our cohort was 24 years old, and the oldest was 98 years old (76.99 ± 13.894). The BMI ranged from 18.03 to 39.79 (24.98 ± 4.74). The median ASA score (assessing patients’ fitness before surgery) was 3 (range: 1–4). A total of n = 6 patients were treated with the Percusys^®^ screw-rod system (joimax^®^ GmbH), whereas n = 62 patients received the MIS Z-Pedicle Screw System (Z-Medical). The most common fracture types according to the AO classification were A3 (40.3%) and A4 (40.3%), followed by B2 (7.46%) and B1 (5.97%). The most common index fracture was TH12 (31.71%), followed by L1 (26.83%) and TH11 (9.76%). While index fractures ranged from TH7 to L4, stabilized levels ranged from TH4 to L5 (mean number of targeted levels: 4.25 ± 1.4), with TH10-L2 (12/68) and TH11-L3 (11/68) being the most frequent site of surgery. Three patients had balloon kyphoplasty (two patients at level L2 and one patient at level TH12). One patient had a A3 burst fracture at L1 and was treated with percutaneous stabilization of Th11-L3, nucleotomy at Th12/L1 and L1/L2, and corporectomy and vertebral replacement at level L1 (Figure 1). There was no need for decompression and nucleotomy for any other cases in the same hospital stay. Mean OT and LOS were 92.2 ± 28.2 min and 14.3 ± 6.8 days, respectively. We observed 9/68 complications (13.2%), mostly involving screw misalignments and loosening. Two cases with loosening of the osteosynthesis material required revision surgery. Both revisions were performed after the 1-year follow-up evaluations. CRP increased from 24.9 ± 33.3 preoperatively to 34.8 ± 29.9 postoperatively (*p* < 0.001), whereas leucocyte counts remained stable. Postoperative (1 day after surgery) hemoglobin, hematocrit, and erythrocyte counts were significantly lower than corresponding preoperative values, whereas thrombocyte counts increased (*p* < 0.001). Coagulation parameters did not significantly differ between the preoperative and postoperative time points. Further, there was a significant improvement in renal function parameters in the postoperative assessment compared to the preoperative values. Table 1 summarizes the characteristics of our cohort.

### 3.2. Assessment of PROMs

**Figure 2:**
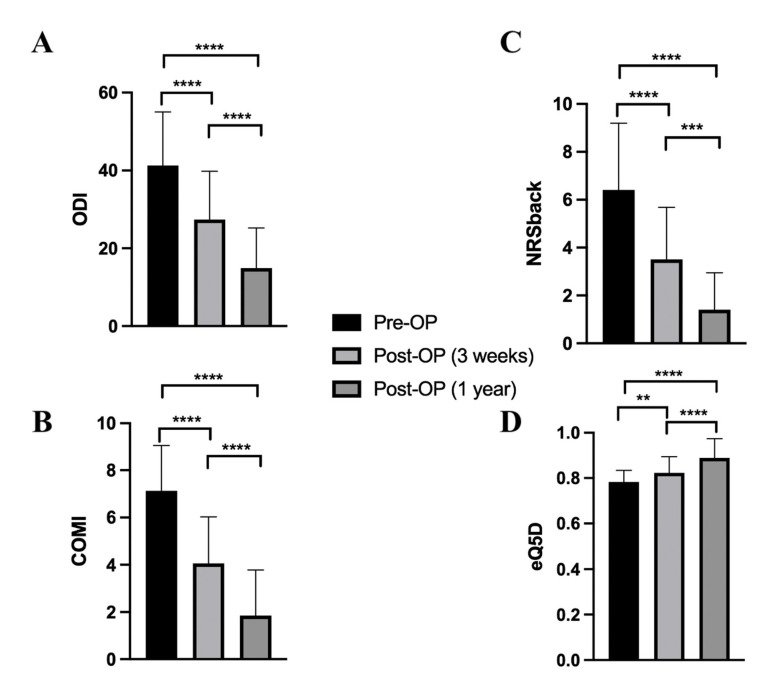
Bar charts show the ODI (**A**), COMI (**B**), NRS back (**C**), and eQ-5D (**D**) difference between time points after thoracolumbar spine stabilization utilizing minimal-invasive percutaneous screw-rod stabilization. Pre-OP: preoperatively; Post-OP (3 weeks): 3 weeks postoperatively; Post-OP (1 year): 1 year postoperatively. Bar charts show the mean ± sd. ** *p* < 0.01, *** *p* < 0.001, **** *p* < 0.0001.

All PROMs showed marked improvements for both postoperative time points (Figure 2). ODI decreased from 41.93 ± 13.75 preoperatively to 27.37 ± 12.40 and 14.94 ± 10.28 after 3 weeks and 1 year postoperatively, respectively (*p* < 0.0001). Comparable to the ODI evaluations, COMI showed a significant decrease from the preoperative situation (7.13 ± 1.93) to the 3-week (4.07 ± 1.96) and 1-year (1.86 ± 1.92) postoperative values (*p* < 0.0001). In addition, there was an increase in eQ-5D from preoperative assessments (0.78 ± 0.05) to the 3-week (0.82 ± 0.07) and 1-year (0.89 ± 0.09) postoperative data (*p* < 0.0001). Further, NRS for back pain showed marked improvements for both postoperative time points.

### 3.3. Analysis of Association with PROMs

Additionally, we dichotomized PROMs in “clinically significant improvement” versus “no clinically significant improvement”, using at least 25% improvement of the postoperative situation as the cut-off value for a minimally important clinical difference to explore significant relationships of study variables utilizing Cramer’s V, phi coefficient, and Pearson’s chi-squared statistics for measuring the associations between the categorical study variables and the dichotomized PROM outcomes [18]. The results are shown in Table 2. We could not find significant associations for most of the analyses. Complication rates showed a strong correlation with the 3-week postoperative COMI (phi coefficient: 0.232), indicating that the complications that occurred negatively affected COMI outcomes after 3 weeks; however, this finding just missed significance (*p* = 0.068). For the 1-year COMI evaluation and the other PROMs, we did not find an association between complication rates and postoperative PROMs. Further, there was also evidence that the ASA score was strongly associated with postoperative NRS evaluated after 3 weeks (Cramer’s V: 0.321). However, also this finding also missed significance (*p* = 0.086). Notably, we found a significant and strong correlation between the reposition level categories and the 1-year NRS evaluations (Cramer’s V: 0.729; *p* = 0.007). Interestingly, more short-spanning stabilizations around Th9-L1 contributed to this finding compared to more long-level stabilizations (e.g., TH6-L3 or TH9-L2). A subsequent pairwise comparison of the number of levels stratified by the dichotomous outcome of NRS (“clinically significant improvement” versus “no clinically significant improvement”) where the cut-off was set as a minimal improvement ≥ 2 in the NRS score confirmed this indicative finding and showed that the number of levels was significantly different between these two groups. The “clinically significant improvement” group had significantly more levels treated compared to the “no clinically significant improvement” group, indicating that more stabilized levels led to better improvements in NRS back pain evaluations after 1 year. However, this finding was only evident for the NRS back pain 1-year evaluation and was not found for the other PROMs, limiting the clinical interpretation.

We further performed logistic regression analyses for each of the PROMs and postoperative time points utilizing the dichotomized PROMs as the dependent variable. We did not find significant associations for the ODI assessments. For the 3-week COMI assessments, we found a significant association for LOS (OR: 0.81; 95% CI: 0.699–0.946; *p* = 0.007), complications (OR: 0.03; 95% CI: 0.001–0.494; *p* = 0.015), and age (OR: 1.19; 95% CI: 1.02–1.38; *p* = 0.027). These findings indicate that a higher LOS and complication rate and a lower age were associated with worse COMI outcomes after 3 weeks in our cohort. However, these findings were not evident anymore for the 1-year COMI evaluations. Additionally, we found a significant association between the number of levels targeted and the NRS back pain evaluations after 1 year (OR: 2.68; 95% CI: 1.11–6.46; *p* = 0.028). This finding follows the results shown in Table 2 and indicates that a higher number of targeted levels was associated with better NRS back pain outcomes after 1 year. 

## 4. Discussion

The present study aimed to evaluate patient-related outcome measures of patients having thoracolumbar fractures treated with percutaneous stabilization utilizing minimal-invasive screw-rod systems. We focused on a range of study variables to examine their association with PROMs. The findings revealed satisfactory outcomes for up to 1 year of follow-up. Further, we found that a higher number of levels treated is not an adverse indicator for PROMs. Our results also showed that a higher duration of hospital stay and a lower age was significantly associated with impaired COMI outcomes after 3 weeks. Notably, PROMs findings were not consistent, leading to the recommendation to assess multiple PROMs in clinics to avoid missing adverse PROMs that are probably relevant for adjusting treatment strategies.

### 4.1. Length of Stay

Percutaneous dorsal stabilization is presently the workhorse in thoracolumbar spine surgery. Compared with open reduction techniques, the reported advantages are shortened operative times, sparing of soft tissues, lower intraoperative blood loss, and reduced postoperative morbidity [22,23]. Two studies that examined the duration of surgery patients operated percutaneously for unstable thoracolumbar fractures reported a range of 60–122 min [24,25]. In accordance with this data, the operation time in our study was 92.21 ± 28.19. Notably, the operation time depends on the surgeon’s experience and might be different in the early implementing phase. Our data included all patients treated with this procedure since inception, and the surgeon’s learning curve is therefore included in our outcome evaluations. Future studies might consequently report shorter operation times for the minimal-invasive approach. Mean LOS in our study following percutaneous treatment of fractures was 14.31 (±6.81) days. Mean hospital stay reported by a meta-analysis of 12 studies that considered 279 percutaneous procedures ranged from 7.6 ± 3.8 days to 12.9 ± 5 days [26]. The meta-analyses reported a mean decrease in hospital length of stay of 5.72 days when compared to the open surgical group. Although our mean hospital stay was higher than the values reported in this meta-analysis, some patients in our cohorts had high hospital stays, which skewed the data, leading to higher standard deviations than those found in the aforementioned studies. When considering the median and the interquartile range, the hospital stay data is better fits the upper range of hospital stay values reported in the literature (median: 12.5; IQR: 10–18). Notably, PROMs or their clinical, laboratory, and radiological predictors are not the only factors affecting the hospital stay: the characteristics of the healthcare system, insurance status, and hospital policies also have an effect, making it difficult to compare the hospital stays from different studies [27,28].

### 4.2. PROMs 

Our analysis of associations between PROMs and relevant study variables showed that long-segment stabilizations were associated with better NRS back pain results after 1 year than short-segment stabilizations. Sapkas et al. [29] focused on the treatment of unstable thoracolumbar burst fractures and revealed that long-segment stabilization is associated with better results with regard to radiological parameters and the Low Back Outcome Score. This was also shown in a meta-analysis [30], concluding that long-segment fixation is associated with better results when radiographic indexes and failures are concerned. However, clinical outcomes assessed did not differ between the short-segment and long-segment pedicle screw stabilization groups in this meta-analysis. Our data also only showed significant associations for the 1-year NRS back pain assessments. Notably, these data assessed open reduction techniques, and our results are only based on the minimal-invasive percutaneous technique. Based on the evidence provided by other authors, long-segment fixation offers greater stability and a more effective reduction in kyphotic deformities [31,32]. Long-segment fixation involves a greater number of targeted vertebrae, which significantly extends the length of the immobile segment in the spine. Several authors recommend three-level stabilization (two levels above and one level below the fracture level) for fractures at the thoracolumbar region, in combination with short-segment fixation in the lumbar area to allow sufficient spinal stability with a reduced length of the immobile segment, leading to a potential reduction of kyphotic collapse rates [33,34,35]. Interestingly, our analysis revealed that lower age was significantly associated with worse COMI outcomes after 3 weeks postoperatively. This is contrary to other findings which show no significant association [36] or an inverse association between age and PROMs after spine surgery 51–54 [37,38,39,40]. However, our data is in accordance with the findings of one of the largest predictive studies in spine surgery, involving 15 hospitals and 1965 surgical patients [41]. The authors showed that lower age was associated with lower odds of PROMs improvement. Similarly, McGirt et al. showed that older age had greater odds of resulting in better PROMs [42]. Thus, in accordance with our data, both showed a positive and non-inverse relationship between PROM improvements after surgery and age, which is counterintuitive. However, this was only present for the COMI outcomes in our study. Notably, satisfaction is a complex and multifactorial outcome measure, which depends not only on the multimorbidity of patients but also on life experience and socioeconomic status [43]. One explanation for the association between PROMs and age after orthopaedic surgery was provided by Baker et al. [44]. Based on a registry-based analysis of 8231 patients, they concluded that patients aged < 65 years were more likely to be dissatisfied than those aged 70 to 80 years with respect to PROMs. Considering these controversial data and our retrospective study design, prospective randomized controlled trials are warranted to better explore the association of age and PROMs with consideration of a broad range of potential confounding factors. Our results also showed that the length of hospital stay and complications were significantly associated with worse COMI outcomes after 3 weeks, but PROMs were not significantly associated with the complication rates after 1 year. Interestingly, these findings were also observed by other authors [45,46,47,48]. Similar to our results, Bortz et al. found that complications did not correlate with PROMs in 182 patients undergoing thoracolumbar surgery [48]. In contrast to the present data, these studies found significant associations between ODI and length of hospital stay, which was also found in one of our previous studies [49]. ODI and COMI are usually highly correlated [50]. Future studies with larger cohorts analyzing this association might come to more consistent results for the ODI and COMI associations with the length of hospital stay.

### 4.3. Laboratory Data

Other outcome predictors often reported in studies comparing minimal-invasive techniques with open surgery in spinal procedures are the CRP and creatinine kinase activity as soft tissue damage markers [51,52]. Whereas creatine kinase is used to quantify tissue damage, CRP is used to quantify associated inflammatory reactions [53]. Unfortunately, we were not able to assess the creatine kinase levels as these are not regularly recorded in patient electronic health records. Grass et al. [54] compared patients with open and percutaneous dorsal spondylodesis using electromyography measurements and found iatrogenic damage to the posterior spinal nerves and muscle fibers of the multifidus muscle from the surgical approach, with significantly higher blood loss in open procedures. Accordingly, animal models showed reduced postoperative creatine kinase levels with the percutaneous approach [55]. The increase of CRP observed in our cohort after the procedure on the first postoperative day was just over 40%. This finding does not follow other reports, where CRP increases of more than 10-fold were reported for this procedure [51,56]. Alterations in CRP levels are known to be associated with surgical trauma, and peaks are usually observed after 48 h postoperatively [57,58]. Nevertheless, the CRP alterations as a response to iatrogenic traumatic injury are highly variable and dependent on numerous patient characteristics [57] and may even be absent in some patients [59]. Thus, comparisons of CRP levels between studies might be limited, and outcome interpretations and comparisons of the techniques should rely more on PROMs than on surrogate markers such as CRP. Nevertheless, they can help to identify adverse outcomes, and CRP levels can be used as one quantifying parameter for the degree of surgical trauma [60], although there are also controversial statements in this regard [61]. Notably, we also found significant differences in other laboratory data. Hemoglobin, hematocrit, thrombocyte counts, erythrocytes, creatinine, and urea significantly decreased after surgery, whereas glomerular filtration rate increased. However, as we did not compare different surgery treatment groups, so it is not possible to provide a comparative analysis of these values. Furthermore, to the best of our knowledge, there are no studies providing these blood and renal function markers in a similar study design for comparison. Therefore, the interpretation of these values is limited. Based on our experience, blood value changes are considered typical during the stabilization of a fractured spine and are therefore not regarded as pathological. The provided laboratory data can help future studies to compare their results with the present findings. In addition, systematic reviews and meta-analyses could use these values for comparisons of different studies. Thus, it is important to report these laboratory values. In a study by Chung et al., the mean intraoperative blood loss was almost five times lesser in the minimal-invasive stabilization group than in the open surgery group [62]. Literature showed that open surgery is associated with increased muscle damage, which might contribute to more blood loss during surgery [63]. Ganse et al. measured blood oxygenation, haemoglobin concentration, and blood flow at different depths and locations on the skin after open versus minimal-invasive posterior stabilization of thoracolumbar fractures [64]. They found higher blood flow values for the minimal-invasive procedure and concluded that skin tissue is spared compared to the open operation.

### 4.4. Other Considerations 

Recently, Erichsen et al. [65] assessed PROMs of patients treated with percutaneous versus open posterior stabilization in AOSpine type A3 thoracolumbar fractures and reported that there was no difference in PROMs for at least 24 months of follow-up between both techniques. Furthermore, reduction loss at the time of follow-up was significantly lower for the minimal-invasive approach. Palmisani et al. [66] used percutaneous techniques as an alternative to the conservative approach and reported satisfactory outcomes with regard to radiographic evaluations of the segmental kyphosis and wedging deformity of the involved vertebral body. Although no PROMs were assessed in this study, and considering that higher kyphotic curvatures are not necessarily related to PROMs [67], the distribution of fracture types was similar to that of the present study, indicating that the procedure is especially safe for relatively stable type A fracture types that failed to improve in response to conservative therapies. With regard to screw positioning, there is little data to date for percutaneous and open procedures. Grass et al. [54] found screw malposition in 6% with percutaneous instrumentation and 12% with an open approach. Another study revealed a screw malposition rate of 2.2% in 685 post-study screw positions [68]. In our study, we used navigation and 3D imaging to place the screws. Notably, intraoperative navigation and the learning curve of the surgeon might affect the prevalence of malposition and, thus, affect comparison between different institutes. 

It can be summarized that percutaneous techniques are increasingly being used, and in our opinion, after the plateau of the learning curve has been reached, they represent a safe procedure with significant advantages over open procedures. In addition, current evidence suggests that the minimal-invasive approach is associated with cost savings when compared to open surgeries [56]. In the literature available to date, it remains unclear whether grossly dislocated fracture forms with segmental kyphosis and/or concurrent axial deviation in the frontal plane are suitable for minimal-invasive dorsal stabilization procedures. There is general agreement that a percutaneous approach should not be used at the expense of insufficient fracture reduction [23,68]. In this regard, Blattert et al. [69] suggested that if spontaneous correction of the deformity by positioning on the operating table is inadequate, open reduction using the righting leverage effect through the pedicle screws should be undertaken. Data from Lendemans et al. [23] showed that reduction using percutaneous procedures in some cases resulted in an incomplete reduction without complete compensation of segmental kyphosis. Wang et al. [70] found a significantly worse recovery of the ventral column to 90.1% of the normal vertebral body height and a loss of correction within 6 months of 3.2 degrees with a percutaneous procedure compared to a correction to 95.8% and 3.0-degree loss of correction with open reduction. However, in this study, patients with higher kyphosis were openly reduced and instrumented, while patients with lower axial deviation were percutaneously reduced and instrumented. In summary, there is little consensus, particularly on the limitations of minimally invasive procedures for more unstable, dislocated fractures; one limitation of percutaneous procedures is an incomplete alignment of the fracture [22,68]. A minimal-invasive reduction is now attempted in most thoracolumbar injuries in our clinic. In these cases, sufficient percutaneous fracture elevation is achieved with the described percutaneous stabilization technique. According to Palmisani et al. [9], a certain loss of reduction may occur, especially in more unstable fracture types; thus, another intervention might be required after the follow-up interval. As the 1-year-examination was the last available time point as part of our study design, we cannot rule out complications and re-interventions required after the 1-year interval.

### 4.5. Strengths and Limitations

There are certain strengths and limitations associated with the present study. In contrast to most available studies on this topic comparing open versus minimal-invasive reduction spine surgery, we assessed associations and predictive factors for PROMs in patients treated with percutaneous stabilization of thoracolumbar fractures. The findings could help to improve the clinical knowledge with regards to the factors affecting patient outcomes in spine surgery, potentially leading to more accurate predictive models in the future. These models have clinical and scientific relevance, as their results will be helpful for scientists designing appropriate studies and reporting these relevant predictive variables for future evidence-summarizing meta-analyses. This would lead to more reliable comparisons and evaluations of available evidence regarding surgical techniques, improving patient outcomes in the future. In particular, missing predictive studies would increase the difficulty of the process of finding relevant variables to include and report in the study designing process. Study limitations include the retrospective design, which is associated with a lower evidence grade than randomized controlled trials comparing the open reduction technique with the minimal-invasive approach. In addition, our data were obtained within a monocentric study. Multicentric studies could better address the variability available between different institutions, leading to better generalizable predictive results. Furthermore, our study might be associated with selection bias, as we had to use the data as provided in our patient information system without further validation, and additional consultations to extract missing data in some variables are often not possible. Moreover, we could not include other variables which could be relevant, such as interleukin-6 or creatine kinase as a surrogate marker for tissue damage, as these are not regularly measured in our clinics and thus not available in the patient information system. Therefore, well-designed randomized controlled studies focusing on a broad range of relevant outcomes and considering relevant predictive factors within their study design are warranted to improve the knowledge currently available in the literature.

## 5. Conclusions

Minimal-invasive stabilization of thoracolumbar fractures utilizing a dorsal percutaneous approach resulted in significant PROM outcome improvement for up to 1 year of follow-up. However, we found a complication rate of 13.2% in our cohort. Increased postoperative CRP levels did not seem to have clinical relevance. Furthermore, we found that a higher number of levels treated and a higher complication rate are not adverse indicators for PROMs. Overall, PROMs were not significantly associated with clinicopathological characteristics, technique-related variables, or the number of targeted levels. More studies focusing on factors affecting PROMs in patients treated with percutaneous stabilization of thoracolumbar fractures are warranted for more accurate predictive models and outcome assessments.

## Figures and Tables

**Table 1:** Description of clinical and laboratory characteristics in the cohort (n = 68) and pairwise comparisons of preoperative versus postoperative (1 day) laboratory values. Wilcoxon signed-rank test was used for pairwise comparison. Data are presented as mean ± standard deviation.

		**Pairwise Comparisons**		
**Variable**	**Cohort Characteristics** **n = 68**	**Preoperatively**	**Postoperatively**	***p*** **-Value**
Gender				
Female	37			
Male	31			
Age (years)	76.99 ± 13.89			
Body mass index (kg/m^2^)	24.98 ± 4.74			
Number of levels	4.25 ± 1.41			
Screw-rod system				
Percusys	6			
Z-Medical	62			
Screw type				
Polyaxial	62			
Quattroaxial	6			
Complications				
No	59			
Yes	9			
Operation time (minutes)	92.21 ± 28.19			
Length of stay (days)	14.31 ± 6.81			
White blood cell count (per nL)		8.56 ± 3.19	8.78 ± 3.37	ns
C-reactive protein (mg/L)		24.87 ± 33.29	34.84 ± 29.87	<0.001
Hemoglobin (g/dL)		12.74 ± 1.81	11.50 ± 1.80	<0.001
Hematocrit (L/L)		0.37 ± 0.05	0.34 ± 0.05	<0.001
Thrombocytes (per nL)		280.7 ± 92.01	322.9 ± 107.92	<0.001
Erythrocytes		4.15 ± 0.61	3.78 ± 0.55	<0.001
International normalized ratio		1.03 ± 0.086	1.02 ± 0.032	ns
Prothrombin time (%)		94.9 ± 11.98	100.2 ± 15.27	ns
Partial thromboplastin time (seconds)		28.8 ± 3.69	28.6 ± 2.61	ns
Sodium		137.8 ± 4.37	138.4 ± 4.32	ns
Blood sugar level (mg/dL)		115.7 ± 35.30	118.2 ± 25.20	ns
Creatinine (mg/dL)		0.86 ± 0.45	0.82 ± 0.68	<0.001
Glomerular filtration rate (ml/min)		90.30 ± 31.00	105.2 ± 39.02	<0.001
Urea (mg/dL)		38.00 ± 23.90	33.38 ± 31.63	<0.001
ns: not significant				

**Table 2:** Analysis of associations between PROMs and categorical study variables. Interpretations of Cramer’s V and phi coefficient are made as follows: very strong correlation (>0.25), strong correlation (>0.15), moderate correlation (0.10), weak correlation (>0.05), no or very weak correlation (>0). * *p* < 0.05, ** *p* < 0.01.

	**ODI**					
	**3 Weeks Postoperatively**			**1 Year Postoperatively**		
**Variable**	Phi Coefficient or Cramer’s V	Chi-squared	*p*-Value	Phi Coefficient or Cramer’s V	Chi-squared	*p*-Value
Gender	0.127	1.004	0.316	0.009	0.005	0.947
Screw-rod system	0.198	2.419	0.120	0.086	0.458	0.499
Screw type	0.115	0.814	0.367	0.069	0.295	0.587
Complications	0.025	0.038	0.845	0.095	0.557	0.456
ASA	0.2617	3.928	0.269	0.096	0.569	0.904
Reposition Level	0.661	27.08	0.352	0.702	30.519	0.205
	**COMI**					
	**3 Weeks Postoperatively**			**1 Year Postoperatively**		
**Variable**	Phi Coefficient or Cramer’s V	Chi-squared	*p*-Value	Phi Coefficient or Cramer’s V	Chi-squared	*p*-Value
Gender	0.095	0.557	0.455	0.132	1.084	0.298
Screw-rod system	0.197	2.412	0.120	0.042	0.109	0.741
Screw type	0.158	1.553	0.213	0.034	0.070	0.791
Complications	0.232	3.335	0.068	0.049	0.151	0.698
ASA	0.279	4.813	0.186	0.200	2.485	0.478
Reposition Level	0.535	17.769	0.852	0.701	30.492	0.206
	**eQ5D**					
	**3 Weeks Postoperatively**			**1 Year Postoperatively**		
**Variable**	Phi Coefficient or Cramer’s V	Chi-squared	*p*-Value	Phi Coefficient or Cramer’s V	Chi-squared	*p*-Value
Gender	0.101	0.688	0.407	0.079	0.421	0.516
Screw-rod system	0.208	2..940	0.086	0.172	2.011	0.156
Screw type	0.129	1.127	0.288	0.037	0.092	0.761
Complications	0.167	1.899	0.168	0.172	2.017	0.156
ASA	0.247	4.142	0.247	0.282	5.401	0.145
Reposition Level	0.601	24.594	0.597	0.682	31.648	0.245
	**NRS-Back**					
	**3 Weeks Postoperatively**			**1 Year Postoperatively**		
**Variable**	Phi Coefficient or Cramer’s V	Chi-squared	*p*-Value	Phi Coefficient or Cramer’s V	Chi-squared	*p*-Value
Gender	0.199	2.539	0.111	0.289	5.379	0.020 (*)
Screw-rod system	0.260	4.337	0.037 (*)	0.024	0.037	0.847
Screw type	0.066	0.276	0.599	0.049	0.158	0.691
Complications	0.039	0.096	0.756	0.017	0.019	0.892
ASA	0.321	0.606	0.086	0.244	3.809	0.283
Reposition Level	0.729	34.015	0.135	0.855	46.761	0.007 (**)

## Data Availability

Data supporting the findings of this study are available from the corresponding author upon reasonable request.
